# Supplementary material for: Genomic organization of the autonomous regulatory domain of eyeless locus in Drosophila melanogaster
Source: G3 (Bethesda). 2021 Sep 27;11(12):jkab338. doi: 10.1093/g3journal/jkab338 (PMC8664461; doi:10.1093/g3journal/jkab338)
Supplement: jkab338_Supplementary_Data [file jkab338_supplementary_data.pdf]

# Supplementary Figure 1

TGTTTTTGCCCGCGAACGTTTGGCTGGAAAGATTGGGTTGCCAGAGGCAAGAATTCAGGTTTGGTTCTCAAACCGTCGAGCAAAATGGCGTCGCGAGGA
GAAGCTGCGAAACCAGCGAAGAACACCAAATTCCACAGGAGCTAGTGCAACTTCTTCCTCTACATCGGCAACCGCCTCTTTGACTGACAGCCCTAACAG

INT7

CCTAAGTGCTTGTTCCTCGCTGCTGTCCGGATCAGCTGGGGGTCCCTCAGTCAGTAAGTATAAATAATTTTGAA**ATCAAGGACCTTGTCGTGACACTC**
**TTCAAAGTCGCTCTTATGAAAGAGATTACAACATGGTCGTTACATTGCATAAAATATTTTTATACAGTTTGCTATATAGTTTACGCTATAAATAT**
**TATTTAAAACAACTTCCTTTTGTTGTTAGTCTTAATCAATCCACAACAAATTGATGTTGACTCTTTTCTCGGGAGTATTTTGCTTATACATTTTTA**
**ACCGTGCATTAGGTATTATATTTTAAATCCAAAGTTTTTGACACATCAATTGGTTCTGTGGCCGGTTAATCATTTATTCTTAGGCGGGTAAGGTT**
**GCTGGTTAAAGATTCGAGGGCTTCGGCAATTTGCAATTCATCACTGCATCAGAATGTTGCGGCTATATATATTTAGTTTTTGGCACTTTTAACTC**
**TGCCCTCTGAATCAGATCTATGTTTCTGTTACTGCCGCTGCCCCATTATTACCAGTGATATTTGCAAAAGGCTTTATGCCGTCGAGAGCTCTTAT**
**TTATATTTTTAGGCACATCCAGTTTTCTGGTCTTTTATTGTTTTATTCCAAGTGTGGCGTTTAAATGAAAAGATTAAATATTTGTTTGTAGATTTC**
**CTCGATGAGGTCAAATTTAGGTATTCTGGAATGATCCATGAATGTGCTCGGCAGGAGTAAGTAATCATATTGTTATCCGTGTTGTAAGGATTTT**
**GCTCAAAAGCTCTCTAAAGGTGTTAGGCTTTTTGCGTCATTTGTTTTGTAGCTGGGATCGGTGGGACTGGAGGAGGATTGAGTATTTCAGTAAC**
**GCTAAAGGTGAGCCAGATTGTTTCAGAACGTTGCCACATCCATTCCAGGGCGTACAAGCTGCTTGGGTTGGCGTTCCATATCTTGCAGAGAAAT**
**CGACGATAGAGGGCGCCAAGTTCTCTTTGTTGCTTCCCGGAGTATAGATAGTAATCTTAATTGAGATCCTACGTTCGGCATACGAGGTTTTTGG**
**GTTTGGTTAACCCAACTTGGTGCGCTTTTCGTTGTGTCGAGGTTTAATTTATGCATAACTGATCGAATAATACAATAATAATAATACAATAATAC**
**AAATGCTCGCTTTAAGCAGATTCCAAATATTTGCCGCAAACAGCATAGCATTCGCTCTCGTTTTTAATTTGAACTGGCTTTATATGCTTAAAATT**
**ATTCGTCAGATCTATTTACAAAACCAAGCCCTCTGCCAGTTCTTGAATAATCTATAATTTATTGTACTAAGTATGACATCTGATTTTAAATGTGT**
**GACGGCAATTTAAG**AATGACCGTAAAATGCAGATAGATAAATTAATTTTCCTATTCCATATTTAGGTACCATTAATGGCTTATCGTCTCCAAGCACATT
GTCTACTAATGTCAATGCTCCAACGCTTGGCGCTGGGATCGATAGCTCTGAAAGCCCAACACCAATCCCGCACATTCGGCCTAGCTGCACCTCTGACAAT
GACAATGGTCGTCAAAGTGAAGATTGCAGAAGAGTTTGTTCTCCATGCCCACTTGGCGTTGGCGGGCATCAAAATACTCATCATATCCAGAGCAATGGT
CACGCCCAAGGTCATGCACTTGTTCCTGCCATTTCGCCACGACTCAATTTTAATAGTGGTAGTTTCGGCGCGATGTACTCCAACATGCATCATACGGCGT
TATCCATGAGCGATTCATATGGGTAAGCATATTCAGCAACTACAGTTTTCAAATTTTTCAGTCATCCCACTTATACAGTATATTAGCTTGCATAATACTGA
AAAAACTCGTTGATGCTATTGCTGGTAATATATTTAGACGGTAGAATAAAAACTCAAAAGAAAACAACACAAGAGATATGTCAACATACTGACTGATA
CATTTGTCATTATATATTAGGAATTAGAAATTGAATCTGTATTAGTTCGTTGATATGGACTTAAGTCACATTAAGAACTTTTACTTCTAAAGCTCCACTCA
ATTAGGAAGAGTTTTGAACCGTATATAGTTTATATATCCTTGCTTAGATCGAGCAAGGATTAGCAATGTACGTCGGTATTCACGATATCACGGAATCTAT
AAAAGATGGTAGGCATAGACAAGGCACGCAGTTTAAAAATGTAAAAAAGTGAGCCGCTCACTTTTTAAAACATATTATTTTTTCATTTTATTCCTTTTCT
TTCAAATTTTTATCTAAATGCCAAAAAAAATTCTGAAGCTTCACCCTCGCACTCCCACTATTTAAGTAATGAGTCTCTCTTTTTTTTATTGCTAATAACCTCT
AGCCTCTAAAATATCAAACTCACTAGGTATGCATAGGTACATTAATACTTATATGTATATCTCATAGTCTATAACCATGTGAAAGTCAATACCTAAATGG
CTTTATTTTTCAGGGCGGTTACGCCGATTCCGAGCTTTAACCACTCAGCTGTCGGTCCGCTGGCTCCGCCATCGCCAATACCGCAACAGGGCGATCTTAC
CCCTTCCTCGTTATATCCGTGCCACATGACCCTACGACCCCCTCCGATGGCTCCCGCTCACCATCACATCGTGCCG**GGTGACGGTGGCAGACCTGCGG**

EB-u

**GCGTTGGCCTAGGCAGTGGCCAATCTGCGAATTTGGGAGCAAGCTGCAGCGGATCGGGATACGAAGTGCTATCTGCCTACGCGTTGCCACCGC**
**CCCCTATGGCGTCGAGCTCTGCTGCTGATTCAAGCTTCTCAGCCGCGTCCAGTGCCAGCGCTAATGTGACCCCACATCACACCATAGCCCAAG**
**AATCATGCCCCTCTCCGTGTTCAAGCGCGAGCCACTTTGGAGTTGCTCACAGTTCTGGGTTTTCGTCCGACCCGATTTCACCGGCTGTATCTTC**
**GTATGCACATATGAGCTACAATTACGCGTCGTCCGCTAACACCATGACGCCTTCCTCCGCCAGCGGCACATCAGCACACGTGGCCCCGGGAAA**
**ACAACAGTTCTTCGCCTCCTGTTTCTACTCACCGTGGGTCTAGGAACAGACTGGCGATTTGAGCAGAGAAGCACTGCGAAAGGACTATTTACAT**
**AGTTGAATGTATATCTAAAGGAGGCCATAATAAATCGAATTTACATATCTCTTGAAAAATAATGGAGGTTGTAGAAAAATACATTTGTATGTATA**
**AATTATATAGTTCCGCCCATTAAATCCAATCTATAGTGTAGAATAATTGGTGTAAATTAAATGATATAATTTTGACAAATAAAAAGAACAAAATG**
**TTGTTTCCTTAAATTTCCTTATTCTTTTCCTTGGAGTCTTCAAAAAGTAAGTGCATAGTTTGTTTTTCAATTTAAAAATGCTTGATCTGTTAAAAA**
**TTTTCTTAAGAGGGCTTAATAATTTTATTGTATTTAGTGGTTCCTTCGCAAACC**TGATATTAATGCAGTGACTTCAATGGTAAATCTGGCAATTTAA
GATTCCAGGGAAGACATTTTCGAATATAATCTCTTTAAGAAACAATCCATCCCAGCACTTGTTACCCTTTTCAGTGGTAAGCTTTAATGTTCCTTTACAAA
ATCTAAAATTTTAAACAGGAACTGTTCTACAAATTAAAATGAAATATATGAATTATCTGATCAAAAAGTTTTTACGACATCTTTTAAATTCAAATTACAT
CTACAAATTTTTACAAGGACAAGGATTAAAAACGAGCAGATACTTTATCTGGGGTCACAAGGGTTACTCTCTTCTTATTATTATTGTTGTTAACTTCTTAA

EB-i

AATAATTTAATACAAATATCATTAAATCAGTTGGTTTTTGCCAAGGTTAGAAAA**ATCATAAATACGTAATTAAGAAAGACTATACGAAAATTAGTAT**
**ATTAAAAATAAGGGTCATACTTTTTGTGATTTACGTCGCTGCAAAAAAAATATTAACTAAGTATCTTATCTAAATATCTCTCCGTTTTGAAGTTA**
**TTAGTATTGCCCAGAAAACGTTGAGAAAGATTTCTCACACATTTTGATAAAACTAACGAAATACACAGAAACAAGAAAACATTATCCAACACTA**
**CTGAAGTGAAATGACATTAATTACAATATTTATTTATTTGGACACGAACCAATGAACATTTTGACTGTCGGAATCTATCAACGGATACACCAAA**
**GGCAAGGTTAATATAAAATGGTTGGAACAGTAAATACTTTGATAAAACATCCCGAACAAGAATTTTAAATAGTGAAATTATTTTCGATAACGTTT**
**GTAACACATTCGTTACTTGTTTTTACATCGTCAAAAGATGTTTTTTGATTAAACAAAAGTTTATACAAGACGTTTATACAAGAATTCCTGTTTGG**
**AATATTTATTAACATGTTATGAATTAACGGCTAATAATAATTATGAGTGATATGTTTATGCCTTTCTGCTGACTTTTTTAATAAGAAAGATAAAG**
**AAATGTGAGATTGGGCACTAAAGGCCAAGATTCGCTAATATTTCTGGAGTTTCTTACATCCTAACAAATGGTAATTGGAACGATATAAACCCTC**
**ATAATGTACAACCTTCTGGAAAAGAAAGTAAGTCTTTTTTTAAATGGAACTTACGTTTAAAATAAGATGGCGTTTCTACATTCTACATTATACCG**
**ATGAATAAGCGTTCGCCATGTAAAGATTTATAAAATGCATTTATACCTTTATACCATTTTAATAGTTTACTCTGACGACTATGATATATAGTAGT**
**GCTGGGTAAACCTAGGTCAATTTGGAGAATAAGCTCGTTCGTTCGTTTAAAATCATTGAACTAGGTCGTTCATTTAGTTCAATCGTTCATTTTTT**
**AAACTTGCTTAAAGGTGCTGC**GCAAAAAAAGTGTTATTTAATTTATTTCATACAATGCCGTCATTGAAAATTAATATTATTTATTCAATTTATGTCAAA
AACACTTGTACATAATAATAATAAGAAATAATAAAGAGTTTTCAACTATATTGAACTGAGTGAACTATATTGAACTATAAAGACAGGCCTAGTTCGTTC
GTTCAGTTTCGTGAATAGTTAAATTTAGTTCGTTCGTTCACGAACGACACAACACTAATATATAGTAACATATATATATATAGATATAACATATAGATAT
GTATATCGATATCCCAGAAAGATGATGAAATTTCGCGATCACATTCTGACACTAGCTGAGTAATGGGTATCTGATAGTCGCGGAACCCGACTTCTGCTTA
AAAGTGTGGTCTTGCCAGTTTTGGGCGGTTTTAGGCGTGGCAAAAATTTTTTTGGCAAATCGATGAAAAATTATCAAAGCATTTTTTTAAAGTGTGGGC
GTGGCACTTTT

Sequence of *ey* downstream region along with DHS (highlighted in yellow) and predicted boundaries (bold) in 7th intron (INT7), 3’ UTR (EB-u), and intergenic (EB-i).

# Supplementary Figure 2

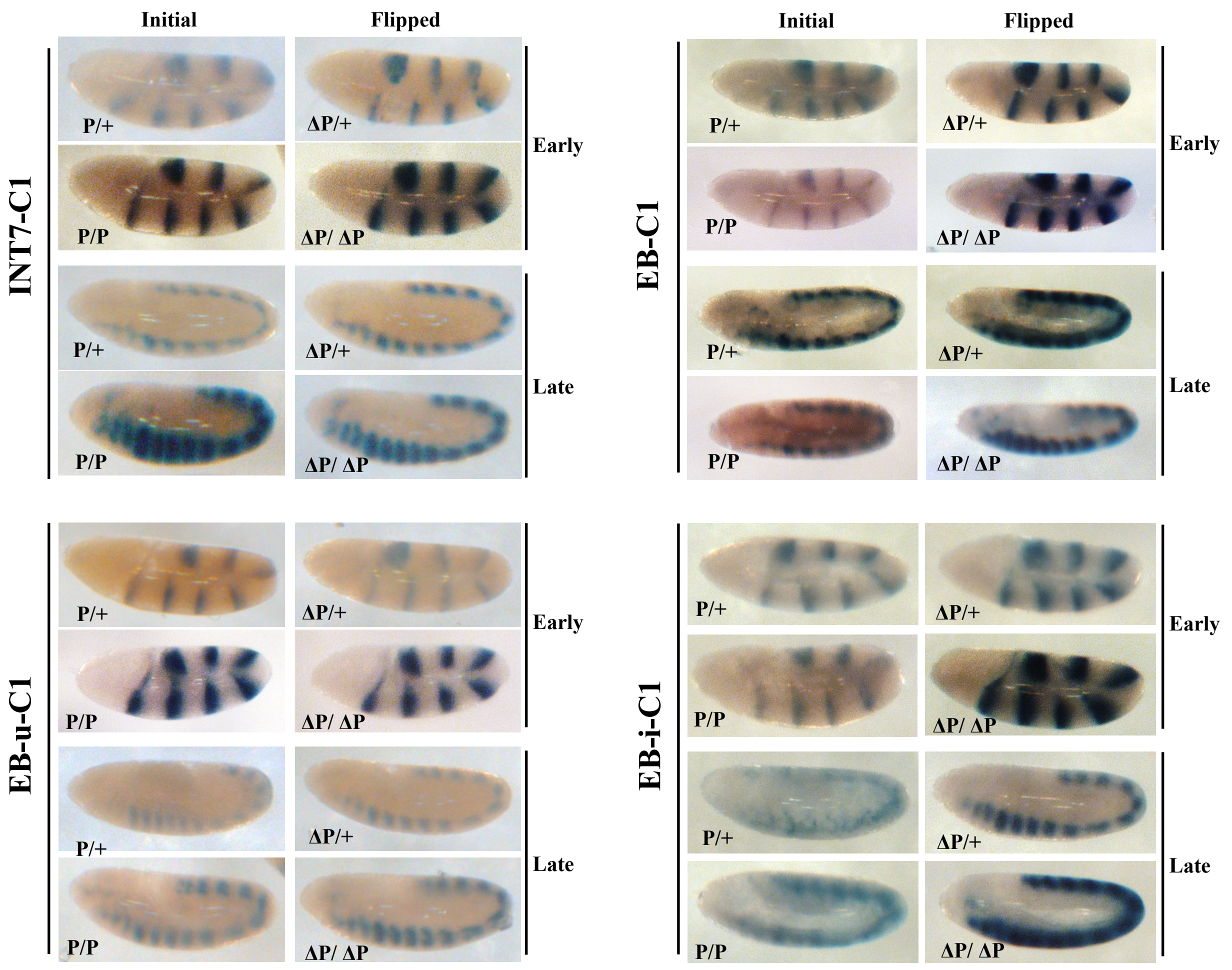


**Enhance blocker assay in embryos.** The figures show LacZ staining in embryos. In INT7 (upper left), a mild increase in staining is seen upon flipping out the test fragment (ΔP) compared to the initial line (P). The enhancer blocker effect is reduced in the homozygous state (P/P), as staining in the initial (P/P) lines is comparable to that in the flipped-out lines suggesting that INT7 possesses a weak boundary activity. EB-u fragment does not show an enhancer blocker effect embryos (lower left). The staining in the initial lines is similar to that in the flipped-out lines indicating an absence of boundary activity. EB and EB-i both show a strong enhancer blocker effect in embryos (right). The staining increases upon flipping out of the test fragment, indicating that EB is an enhancer blocker boundary. The effect is seen in both early and late developmental stages.

## Supplementary Figure 3

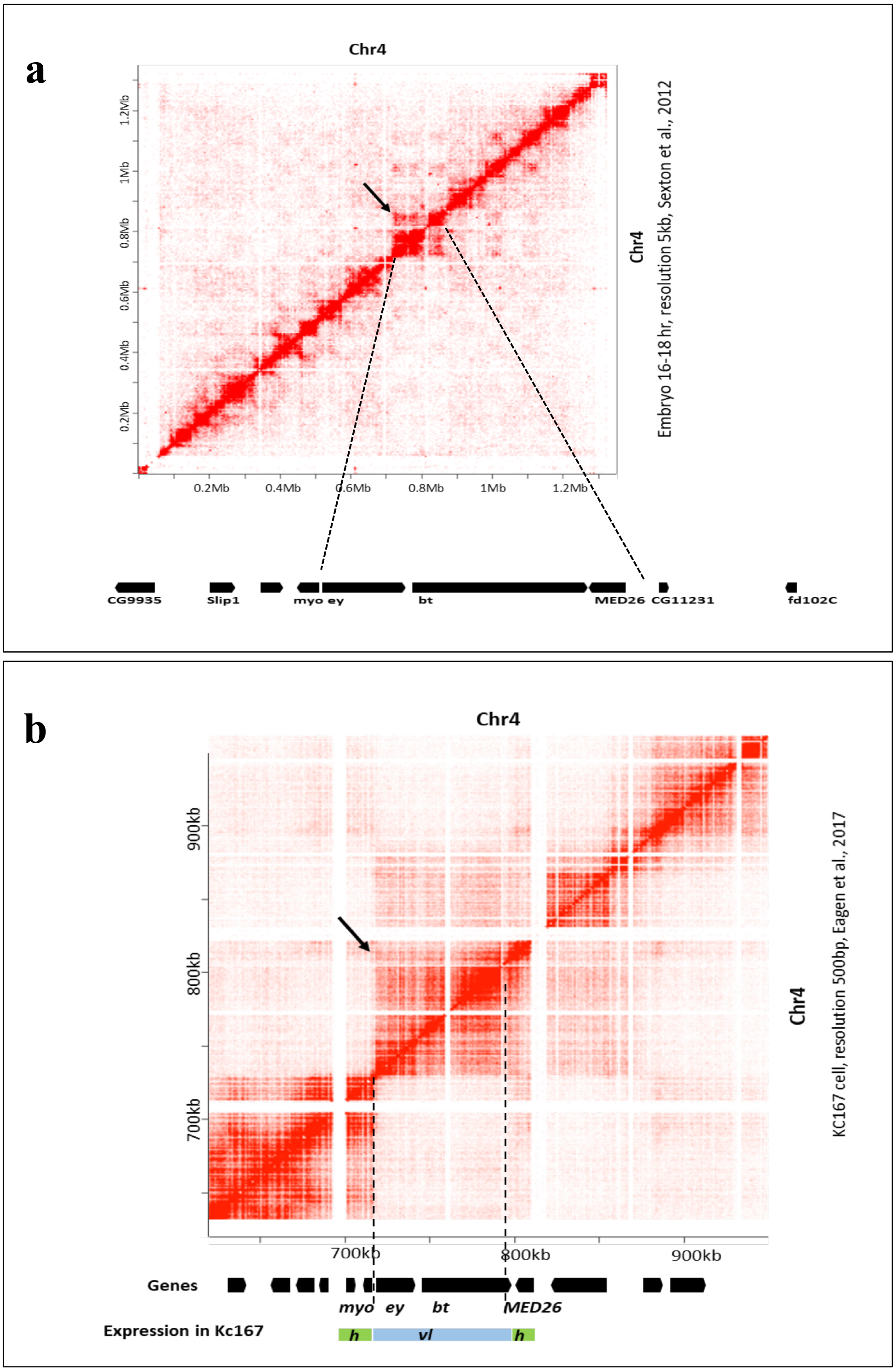


**Long-range interactions at *ey* locus**. (a) Hi-C heatmap of the 4th chromosome in *Drosophila* embryos using juicebox.js from Sexton et al., 2012. The *ey* locus (black arrow) appears to be part of a domain that includes two other genes *bt* and MED26, while *myo* upstream to *ey* is present in a separate region. (b) Chromatin interactions heatmap at *ey* locus (~350Kb) on in *Drosophila* Kc167 cells from Eagen et al., 2017. Two genes, *ey*, and *bt* fall together in one TAD with chromatin contacts within it (vertical dot lines); however, *myo* and MED26 appeared to be part of separate domains. The ME boundary interacts with the *ey*-PRE region. Both the genes, *ey* and *bt,* have a similar and very reduced level (vl) of expression (marked in blue). On the other hand, *myo* and MED26 show a comparatively higher level (h) of expression (marked in green).

# Supplementary Figure 4

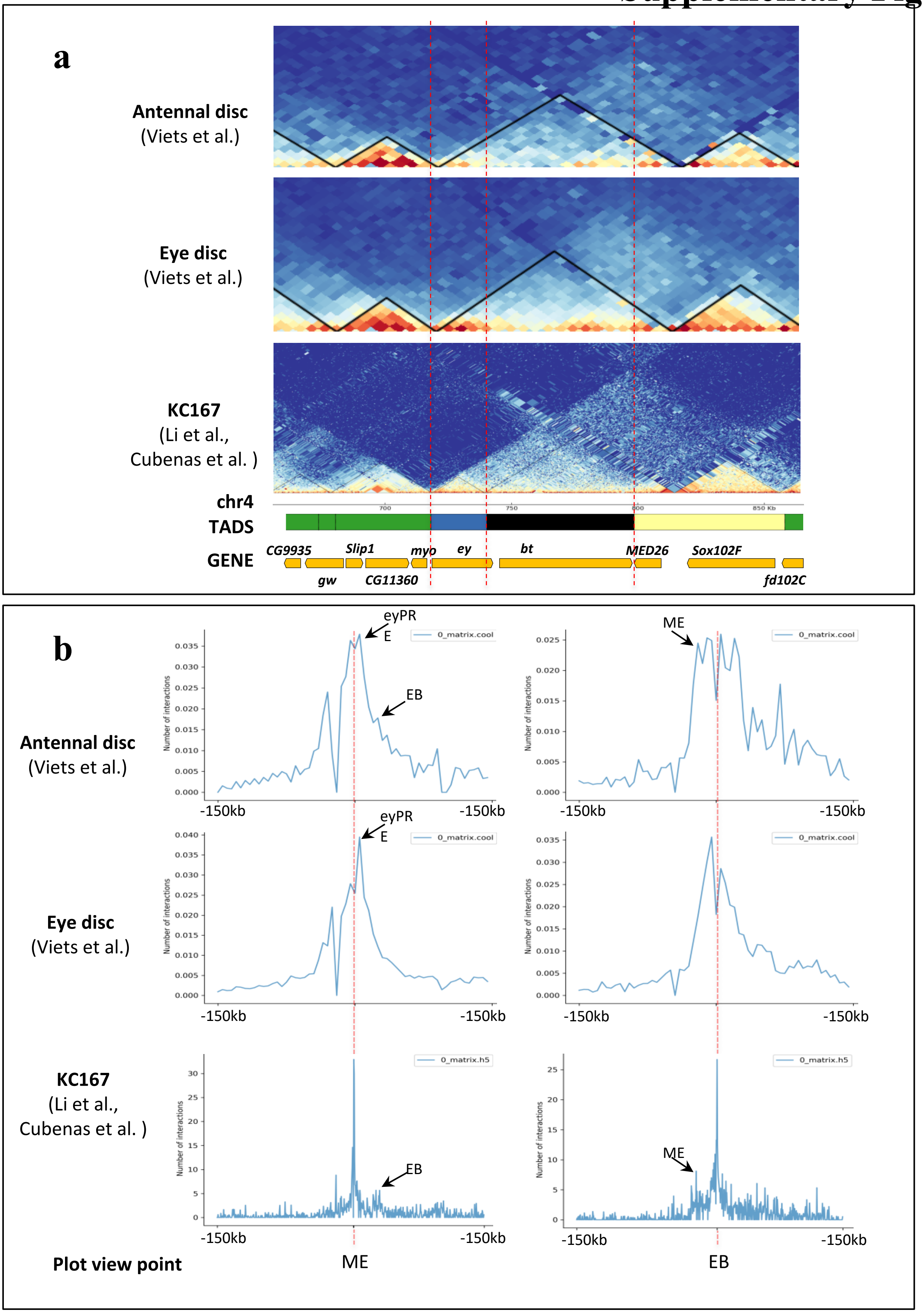


**Comparison of long-range interactions at *ey* locus**. (a) HiC heatmap of the *ey* region in antennal disc, eye disc and Kc167 cells from ref (Viets et al., 2019 Li et al., 2015 and Cubenas et al., 2017). TAD classifications track from ref (red dashed lines) (Ramirez et al. 2018). Heatmap generated using pyGenomeTracks. In all cell types *ey* and *bt* are present in a same *ey*-TAD. (b) Relative chromatin interaction of ME and EB (red dashed lines) to other regions at *ey* locus. Chromatin interactions to 150 kb flanking regions were plotted using Plotveiwpoint in HiC explorer. In all three datasets ME appears to interact with EB (left). Although ME interaction to *ey*-PRE is prominent in eye disc. Similarly, EB appears to interact with ME (right).

# Supplementary Figure 5

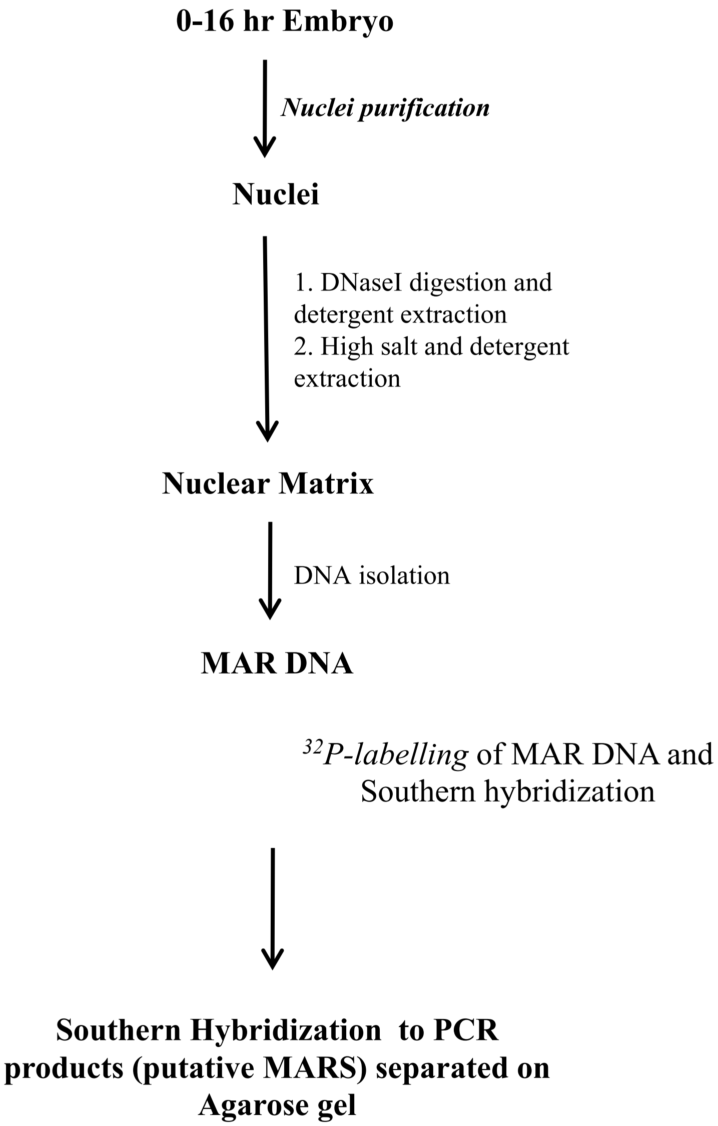


Schematic of *in vivo* NuMat association assay.

# Supplementary Table 1

## List of Fragments tested for boundary and PRE activity

| **Predicted CBEs and PREs** | **4th chromosome Coordinates (dm3)** | **Size (bp)** | **Comments** |
|---|---|---|---|
| INT7 | 738571-739855 | 1285 | 7th Intronic region of *ey* |
| EB | 741132-743358 | 2227 | Intergenic region between ey and bt including 8th exon and 3' UTR of *ey* |
| EB-u | 741132-741979 | 848 | 8th exon and 3'UTR region of *ey* |
| EB-i | 742226-743361 | 1136 | Intergenic region between *ey* and *bt* |
| *ey*-PRE | 724213-725866 | 1654 | Preceding to first exon of *ey* |
| *ey*-dPRE | 724640 -725470 | 831 | Preceding to first exon of *ey* encompass DNaseI hypersensitive sites |

# Supplementary Table 2

## Enhancer blocker activity of tested fragments in adult eye

| Line | Chr | Eye color (P/+) | | Eye color (ΔP/+) | | Comment |
|---|---|---|---|---|---|---|
| | | Male | Female | Male | Female | |
| **INT7 1** | **I** | **Red** | **Red** | **Red** | **Red** | **N** |
| INT7 2 | III | Red | Red | Red | Red | N |
| INT7 3 | I | Bright red | Red | Bright red | Red | N |
| INT7 4 | III | Red | Light red | Red | Light red | N |
| EB 1 | II | Dark orange | Orange | Red | Light red | W |
| **EB 2** | **III** | **orange** | **Light orange** | **Red** | **Red** | **M** |
| EB 3 | III | Red | Orange | Red | Orange | N |
| EB 4 | II | Red | Red | Red | Red | N |
| EB 5 | III | Red | Red | Red | Red | N |
| EB 6 | I | Dark orange | Light orange | Dark orange | Orange | **W** |
| EB 7 | III | Dark orange | Light orange | Dark orange | Orange | **W** |
| **EB-i 1** | **I** | **Light red** | **Orange** | **Red** | **Light red** | **M** |
| EB-i 2 | III | Red | Light red | Red | Red | W |
| EB-i 3 | III | Red | Red | Red | Red | N |
| EB-i 4 | I | Red | Light red | Red | Light red | N |
| EB-i 5 | II | Red | Light red | Red | Red | W |
| EB-i 6 | III | Red | Light red | Red | Red | W |
| EB-i 7 | II | Red | Light red | Red | Red | W |
| **EB-u 1** | **II** | **Red** | **Red** | **Red** | **Red** | **N** |
| EB-u 2 | III | Red | Light red | Red | Light red | N |
| EB-u 3 | III | Red | Light red | Red | Light red | N |
| EB-u 4 | II | Red | Light red | Red | Light red | N |
| EB-u 5 | III | Red | Light red | Red | Light red | N |
| EB-u 6 | I | Red | Light red | Red | Light red | N |
| EB-u 7 | III | Red | Light red | Red | Light red | N |

M-moderate blocker, W-weak blocker, N-non blocker

Transgenic lines in bold were used for pigment assay experiments

# Supplementary Table 3

## Enhancer blocker activity of tested fragments in embryo

| Line | Chr. | Eye color (Male) | | Level of lacZ staining in embryos | | | | Comments |
|---|---|---|---|---|---|---|---|---|
| | | P/+ | ΔP/+ | P/+ | ΔP/+ | P/P | ΔP/ΔP | |
| **INT7 C1** | **II** | **Orange** | **Orange** | + | + + | + + | + + + | **W** |
| INT7 C2 | III | Dark orange | Dark orange | + | + + | + + + | + + + | **W** |
| INT7 C3 | I | Light red | Light red | + + + | + + + | NT | NT | N |
| **EB C1** | **III** | **Light yellow** | **Light yellow** | + + | + + + | + | + + + + | **S** |
| EB C2 | II | Light yellow | Light yellow | + + | **NT** | **-** | + + + | **M** |
| EB C3 | I | Yellow | Yellow | + | + | **NT** | **NT** | N |
| **EB-u C1** | **III** | **Yellow** | **Yellow** | + | + | + + + | + + + | **N** |
| EB-u C2 | III | Yellow | Yellow | + + + | + + + | + + + | + + + | N |
| EB-u C3 | II | Yellow | Yellow | ++ | ++ | **NT** | **NT** | N |
| **EB-i C1** | **III** | **Yellow** | **Yellow** | + + | + + + | + + | + + + + | **S** |
| EB-i C2 | III | Yellow | Yellow | + + | + + + | + + + | + + + + | **M** |
| EB-i C3 | III | Yellow | Yellow | + + | + + | **NT** | **NT** | N |
| EB-i C4 | II | Yellow | Yellow | + + | + + + | + + | + + + + | **M** |

NT- not tested, S-strong blocker, M-moderate blocker, W-weak blocker, N-non blocker

Transgenic lines in bold were used for relative lacZ stainging quantifications

# Supplementary Table 4

## List of *ey*-PRE lines

| S.No. | Line | Chr. | Eye color P/+ | Eye color ΔP/+ | Eye color P/P | PSS | Variegation , comments |
|---|---|---|---|---|---|---|---|
| 1 | 18.12 | III | Light orange | Orange | Orange pattern | No | Yes, patterned eye color |
| 2 | 9.1.2 | I | Yellow | Orange | Lethal | NA | No |
| 3 | 46.1 | III | Light cream | Red | White | Yes | Yes |
| 4 | 23.11 | II | Light Yellow | Orange | Yellow | No | No |
| 5 | 23.1 | III | Orange | Red | Red | No | Yes, homozygous lethal |
| 6 | 35.1 | II | Orange | Orange | NT | yes | Yes |
| 7 | 35.11 | II | Yellow | Orange | Orange | No | Yes |
| 8 | 31.1 | II | Yellow | Orange | Red | No | No, patterned eye color |
| 9 | 88.1 | III | Orange | Red | Yellow | Yes | Yes, variegating only in heterozygous |
| 10 | 88.11 | I | Yellow | Yellow | NT | NT | No |
| 11 | 84.1 | III | Red | Red | Red | No | No |
| 12 | 62.2 | III | Yellow | Red | Red | No | No, patterned eye color |
| 13 | 85.1 | III | Red | Red | Red | Yes | Yes, variegating in heterozygous |
| 14 | 85.11 | I | Yellow | NT | NT | NT | NT |
| 15 | 89.3 | I | Yellow | Yellow | NT | NT | Yes, patterned eye color |
| 16 | 89.13 | II | Red | Red | NT | No | No |
| 17 | 89.4 | II | Orange | Orange, | Red | No | Yes, patterned eye color |
| 18 | 82.12 | III | Yellow | Orange | Orange | No | Yes, variegating only in females |
| 19 | 82.14 | III | Orange | red | Yellow | Yes | Yes |
| 20 | 31.13 | III | Orange | Red | Orange | No | Yes, patterned eye color |
| 21 | 31.11 | II | Yellow | Orange | NT | NT | No |
| 22 | 31.14 | III | Orange | Orange | Orange | No | Yes, variegating in homozygous |
| 23 | 31.16 | III | Light yellow | Dark yellow | L yellow | Yes | Yes |
| 24 | 6.1 | II | Cream | Yellow | Yellow | No | Yes, less variegation |
| 25 | 94.2 | I | Pattern orange | NT | NT | NT | Yes, variegating very peculiar |
| 26 | 94 | II | Pattern | NT | White | Yes | Yes, no pattern in homozygous |
| 27 | 7.2 | I | Yellow | Orange | NT | NT | NT |
| 28 | 57.1 | III | Red | NT | Red | No | No |
| 29 | 64.1 | III | Orange | Orange | Orange | No | Yes, less variegation |
| 30 | 64.1 | I | Red | Red | NT | NT | NT |
| 31 | 97.1 | I | Cream | Orange | NT | NT | No, very light eye color |

## List of *ey*-dPRE lines

| S.No. | Line | Chr | Eye color P/+ | Eye color ΔP/+ | P/P | PSS | Variegation , comments |
|---|---|---|---|---|---|---|---|
| 1 | 10.13 | II | Cream | Red | White | Yes | No |
| 2 | 10.1 | III | Red | Red | Red | No | No |
| 3 | 28.1 | II | Red | Red | Lethal | NA | No |
| 4 | 28.12 | III | Orange | Red | Red | No | No |
| 5 | 28.14 | II | Red | Red | Red | No | No |
| 6 | 25.4 | I | Orange | NT | NT | NT | No |
| 7 | 25.1 | I | Light yellow | NT | NT | NT | No, patterned eye color |
| 8 | 25.5 | III | Light yellow | Yellow | NT | No | No |
| 9 | 18.12 | III | Yellow | Orange | Lethal | NA | No, patterned eye color |
| 10 | 25.7 | III | Orange | NT | Yellow | Yes | Yes |
| 11 | 13.2 | I | Yellow | Yellow | NT | NT | Yes |
| 12 | 13.13 | III | Red | Red | Red | No | No |
| 13 | 13.11 | II | Yellow | Yellow | Yellow | No | No |
| 14 | 47.1 | I | Orange | NT | NT | NT | NT |
| 15 | 47.12 | III | Yellow | Red | Yellow | No | No |
| 16 | 47.21 | I | Yellow | NT | NT | NT | NT |
| 17 | 62.1 | II | Red | NT | Red | No | No |
| 18 | 100.2 | II | Light yellow | Red | White | Yes | Yes |
| 19 | 100.1 | III | Red | Red | Red | No | No |
| 20 | 2.12 | I | Cream | NT | NT | NT | NT |
| 21 | 76.1 | I | Yellow | Yellow | NT | NT | NT |
| 22 | 72.1 | III | Yellow | Orange | White | Yes | No |
| 23 | 49.1 | I | Red | NT | NT | NT | NT |
| 24 | 49.11 | I | Yellow | Orange | NT | NT | NT |
| 25 | 7.1 | I | NT | NT | NT | NT | NT |
| 26 | 10.2 | III | Yellow | Orange | White | Yes | Yes |

NT- not tested

# Supplementary Table 5

| S.No. | Data | Cell type | Online source | Accession Number | Genome Version | Reference |
|---|---|---|---|---|---|---|
| 1 | Chip-chip data: BEAF32, CP190, CTCF and GAF | Embryo (0-12 h) | modENCODE | - | Dm3 | NEGRE ET AL. 2010 |
| 2 | DNAse H.S. stage 9 | Embryo (~ 6 h) | UCSC genome browser data | - | Dm3 | THOMAS et al. 2011 |
| 3 | ChIP-seq data: H3K27Ac (4120), H3K27Me3 (3955), Pc (3957), dRING (5071), Psc (3960), BEAF-32 (3954), CP190 (3959), CTCF (5069), and GAF (4149) | Embryo (14-16 h) | by Gary Karpen at modENCODE | - | Dm6 | MOD et al. 2010 |
| 4 | HiC data, DpnII | Embryo (16-18 h) | Aiden Lab (Juicebox) | GSE34453 | Dm3 | SEXTON et al. 2012 |
| 5 | HiC data, DpnII | Kc167 | Aiden Lab (Juicebox) | GSE89112 | Dm3 | EAGEN et al. 2017 |
| 6 | Merged HiC data, DpnII | Kc167 | Chorogenome data_sources (http://chorogenome.ie-freiburg.mpg.de) | - | Dm3 | LI et al. 2015; CUBENAS-POTTS et al. 2017; RAMIREZ et al. 2018 |
| 7 | HiC data, DpnII | Ey and antennal disc (3rd instar larva) | NCBI | GSE136267 | Dm6 | VIETS et al. 2019 |
| 8 | TAD classification | Kc167 | Chorogenome data_sources (http://chorogenome.ie-freiburg.mpg.de) | - | Dm3 | RAMIREZ et al. 2018 |
| 9 | Chromatin states | Kc167 | NCBI | GSE22069 | Dm3 | FILION et al. 2010; |
| 10 | Lam-DamID | Kc167 | NCBI | GSM509085 GSM509085 | Dm3 | FILION et al. 2010; |

# Supplementary Table 6

**Primers for Boundaries, PREs and NuMat Assay**

| Amplicon | Primer | sequence |
|---|---|---|
| INT7 | INTrF | CGTGAATTCAAAGGACCTTGTCG |
| | INTrR | CACCAAGCTTAAATTGCCGTCACAC |
| EB | UTRrF | CCGGAATTCCTAGGCAGTGGCCAATCT |
| | UIGrR | GAAGATCTCGCAGCACCTTTAAGCAAG |
| EB-u | UTRrF | CCGGAATTCCTAGGCAGTGGCCAATCT |
| | UTRrR | GGAAGATCTAGGTTTGCGAAGGAACCAC |
| EB-i | IGrF | GAAGATCTGGGGTCACAAGGGTTAC |
| | IgrR | CCCAAGCTTTCTGCAGCACCTTTAAGCAAG |
| ey-PRE | EY_PREF | TCACTTTTCAGAAGTCATAAGGACG |
| | EY_PRER | TCTATCAATGTTCCCGCTGAC |
| ey-dPRE | EY_PREFD | TCGATATGGCTGTGTGTGTAGG |
| | EY_PRERD | ACGTATTATTCCCGGGTCTACC |
| HIS MAR | HISMAR_F | TCATATTCGATGATTGGTGGTTG |
| | HISMAR_R | GTACATGTAGGTTTTTAGCTTAGCC |
| BEAF CDS (pBSK clone) | M13F | TGTAAAACGACGGCCAGT |
| | M13R | AGGAAACAGCTATGACCAT |

**Primers for 3C**

| Amplicon | Primer | sequence |
|---|---|---|
| myo-E3 | eylfrg_-3L | TTAGTTAGTCGGTTACACATCT |
| myo-E2 | eylfrg_-2L | CAGGGCTTAATATTGGCTAC |
| myo -E1 | eylfrg_-1L | TTTATAATACAATGTGAGTTGCG |
| eyE1 | eylfrg_0L | AATGCTTCGCTGGTCAT |
| eyE2 | eylfrg_+1L | CGGGTAGTTAACGGAAATGT |
| eyE3 | eylfrg_+2L | AACATGAGTGCAATCAAAAGTG |
| eyE4 | eylfrg_+5L | ACATTCCGCCTAATTTTTCC |
| eyE5 | eylfrg_+6L | TTGAACGAACACACTATCC |
| eyE6 | eylfrg_+7L | TTCGATAACGTTTGTAACACA |
| btE1 | eylfrg_+8L | ACATGCAATACATGAATTTCG |
| btE28 | eylfrg_+9L | GGCGTCGCAACTATTAATCT |
| myo-D2 | Myo1-Dpn-R | GTAGTTGTCACATGTGGGTCATA |
| Myo-D1 | Myo2-Dpn-R | CAGACTGTTCACATAGAAATTGGG |
| eyD1 | ME-1-Dpn-R | CAATGCGTTTCAGCAGATACAT |
| eyD2 | ME-3-Dpn-R | GTCCTTAACAGCGATAAATGTAAGC |
| eyD3 | PRE-1-Dpn-R | TGTAGCAAATCTGAAAGAATTGGG |
| eyD4 | PRE-2-Dpn-R | CGGTGAATCGAACAACCTTTC |
| eyD5 | PRE-3-Dpn-R | CCGAGTCAAGTTAGCGATGT |
| eyD6 | PRE-4-Dpn-R | TGAAGTGTCATCGCCTGTAAA |
| eyD7 | EB-1-Dpn-R | GAAGAGAGTAACCCTTGTGACC |
| btD1 | EB-3-Dpn-R | GCCGATATTATCATGCAGCTCT |
| btD2 | EB-4-Dpn-R | AGCCGCTTTCGAAATTAAGATT |

# Supplementary Table 7

| S.No. | Name | Mutation |
|---|---|---|
| **1** | *CS* | Wild type |
| **2** | *white* | $w^{1118}$ |
| **3** | *Pleiohomoeotic* | $Pho^{1}$ |
| **4** | *Addition sex comb* | $Asx^{XF5}$ |
| **5** | *GAGA factor* | $Trl^{R85}$ |
| **6** | *Polycomb* | $Pc^{1}$ |
| **7** | *Posterior sex comb* | $Psc^{1}$ |
| **8** | *Extra sex comb* | $esc^{2}$ |
| **9** | *Suppressor of zeste* | $Suz2^{1.a1}$ |

| S.No. | Name | Test Fragment | Comments |
|---|---|---|---|
| **10** | CfhL control vector | none | Used as negative control |
| **11** | CfhL *Fab7 vector* | *Fab7* region from the bithorax complex | Used as positive control |
